# The quality of evidence of psychometric properties of three-dimensional spinal posture-measuring instruments

**DOI:** 10.1186/1471-2474-12-93

**Published:** 2011-05-13

**Authors:** Yolandi Brink, Quinette Louw, Karen Grimmer-Somers

**Affiliations:** 1 Division of Physiotherapy, Department of Interdisciplinary Health Sciences, Stellenbosch University, South Africa, PO Box 19063, Tygerberg 7505, South Africa; 2 Division of Health Sciences, University of South Australia, GPO Box 2471, Adelaide, SA, 5000, Australia

**Keywords:** posture measurement, psychometric properties, reliability and validity

## Abstract

**Background:**

Psychometric properties include validity, reliability and sensitivity to change. Establishing the psychometric properties of an instrument which measures three-dimensional human posture are essential prior to applying it in clinical practice or research.

**Methods:**

This paper reports the findings of a systematic literature review which aimed to 1) identify non-invasive three-dimensional (3D) human posture-measuring instruments; and 2) assess the quality of reporting of the methodological procedures undertaken to establish their psychometric properties, using a purpose-build critical appraisal tool.

**Results:**

Seventeen instruments were identified, of which nine were supported by research into psychometric properties. Eleven and six papers respectively, reported on validity and reliability testing. Rater qualification and reference standards were generally poorly addressed, and there was variable quality reporting of rater blinding and statistical analysis.

**Conclusions:**

There is a lack of current research to establish the psychometric properties of non-invasive 3D human posture-measuring instruments.

## Background

Postural assessment is a standard and essential component of examining individuals with neuromusculoskeletal disorders [1,2]. Prolonged static postures are widely recognised as a risk factor of neuromusculoskeletal pain among children, adolescents and adults [3-9]. No uniform definition for "ideal" posture exists and therefore researchers and clinicians continue to seek the best way of assessing and describing posture. Ideal spinal posture is proposed as neutral spinal alignment, however the relationship between spinal segments in a normal population remains unknown [10,11]. The spine is a complex three-dimensional (3D) anatomical structure, whose segmental position in space should be described in all three planes (sagittal, frontal and transverse) [12-14]. Precise positional data can be derived from a number of biomechanical measurement tools, of which non-invasive 3D instruments are preferred.

It is essential that a spinal posture-measuring instrument is shown to be reliable and valid. Without this assurance, it cannot facilitate diagnosis, chart variability in 'usual' posture or assist objective monitoring of patient progress with treatment [1]. Researchers and clinicians should therefore be familiar with the psychometric properties of spinal posture-measuring instruments, and choose the ones with the best evidence of performance [15].

Two core elements of psychometric properties are reliability and validity [16]. Reliability and validity are interlinked of which reliability is a prerequisite to validity. A measurement tool cannot be recommended with confidence if there is a lack of evidence about its reliability and validity [17]. Reliability, refers to being able to estimate the inherent variability of posture, as well as error that can be attributed to the rater and the measurement instrument [17]. Error can relate to the consistency with which measurements are taken by the same or different raters, or over multiple occasions of testing [16]. Reliability is variously classified as test-retest reliability, inter-and intra-rater reliability. Test-retest reliability describes the stability of the measurement instrument in obtaining the same results with repeated measurements using the identical test on two or more separate occasions, keeping all testing conditions as constant as possible [17]. Intra-rater reliability is defined as the stability of data recorded by one observer across two or more test occasions. Inter-rater reliability is the extent to which two or more observers obtain similar scores when rating the same individuals [16,17].

Validity is the extent to which an instrument measures what it is intended to measure [18]. Criterion-related validity is the ability of one test (index test) to predict results obtained on an external criterion (gold standard/reference standard) which is assumed to be valid. When both tests are performed on the same subjects, the scores from the index test are correlated with those achieved by the criterion measure. Construct validity is the ability of an instrument to measure an abstract concept, which cannot be observed directly and which has been constructed to represent an abstract trait [17]. There are two types of criterion-related validity. Concurrent validity is evaluated when the index test and the criterion measure are taken at the same time so that it reflects the same incident of behaviour while predictive validity is tested when the index test is performed and measured prospectively to ascertain the relationship between the index test and the criterion scores to determine whether the index test is a valid predictor of the outcome [17]. There are three types of construct validity. Convergent validity indicates that two measures, which are believed to reflect the same construct, will have similar results or will correlate highly [17]. Whereas divergent validity indicates that two measures, which are believed to measure different constructs, will correlate poorly [19]. Convergent and divergent validity assess the sensitivity and specificity of a measurement respectively [19]. Discriminative validity is the extent to which measures from a measurement instrument distinguishes between individuals or populations that would be expected to differ [19].

Establishing the psychometric properties of spinal posture-measuring instruments is not a trivial task, given the complex nature of human posture. Thus, convincing evidence of reliability and validity of any posture-measuring instrument can only be established by assessing the methodological quality of the underpinning developmental studies. Specific psychometric study design features are therefore essential to establish and assess, for instance, controls that are put in place for systematic bias, non-systematic bias and inferential error. An important requirement for psychometric testing of posture measurement is that the instrument be tested under a given set of conditions on a specific population within the context of the instrument's intended use. Therefore it is essential that posture-measuring instruments be tested on humans at some stage of development, and not just on inanimate objects [17].

The purpose of the systematic review reported in this paper was 1) to identify the non-invasive 3D tools which measure human static sitting or standing spinal posture and 2) to review the quality of the evidence of reliability and validity of the identified 3D posture-measuring instruments.

## Methods

### Search Strategies

Two inter-related search strategies (A and B) were implemented to ensure that all eligible papers were included. Strategy A sought any primary research studies which reported the use of 3D non-invasive instruments measuring static sitting or standing spinal posture. Strategy B sought primary research into the psychometric testing of these instruments. One reviewer searched six electronic databases that were available at the Stellenbosch University Library. The databases were BioMed Central, CINAHL, PEDRO, PROQUEST, PUBMED and SCIENCE DIRECT. The publication date was restricted to papers published from 1980 to June 2010. The search was limited to full-text papers published in English. MESH terms were used in PUBMED. See additional file 1 for a detailed description of the database searches.

In addition, secondary searching was performed on the reference list of the included papers. Experts in this field of research, and authors who failed to provide references to studies which tested an instrument's psychometric properties, were contacted.

### Keywords and synonyms

The following keywords were used: three-dimensional, measurement tool, assessment tool, instrument, measurement, assessment, spinal posture, posture, validity, reliability, accuracy and reproducibility.

### Inclusion and exclusion criteria for selection of papers

Papers were included if they reported testing an instrument's psychometric properties, specifically reliability and/or validity, using humans, or the instrument's validity using objects. A core inclusion criteria was that static standing or sitting spinal posture had to be evaluated with an instrument that could quantitatively calculate 3D spinal posture without using a baseline reference value such as zero. This was because a reference value requires that the subject be required to first assume a neutral or resting posture at which point the instrument is zeroed before the instrument can measure static spinal posture. For the purpose of the review, static posture should be assessed instantaneously without any guiding from the researcher.

Papers were excluded if (1) they reported neither reliability nor validity testing (2) they did not report on static spinal posture (e.g. reported on the 3D motion of the spine, scapulo-humeral girdle or pelvis); (3) the study reported on the validity testing of an instrument using motion (as motion was not incorporated in this review, and we argue that validity be evaluated within the context of the instrument's intended use; (4) the instrument only measured cadaver or in vitro spinal posture; (5) the instrument was invasive e.g. biplanar radiography and stereoradiography; (6) only an algorithm or a mathematical formula were reported.

### Study selection

**Figure 1:**
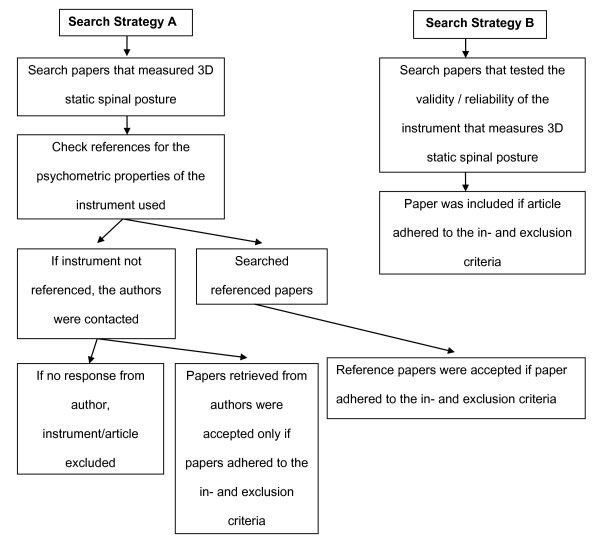
**A Flowchart to demonstrate the procedures for study selection**.

One reviewer excluded papers by screening all the titles and reading the abstracts after which two independent reviewers selected the eligible papers after reading the full text version of the remaining papers. Figure 1 describes the procedures of study selection for each of the two search strategies.

### Methodological Quality Appraisal

The full text eligible papers were then subjected to methodological critical appraisal. The Critical Appraisal Tool (CAT) applied in this review was purpose-built, in the absence of any other relevant CAT. It was adapted from the Quality Assessment of Diagnostic Accuracy Studies (QUADAS) [20] and the Quality Appraisal of Reliability Studies (QAREL) [21]. The purpose-built CAT has 13 items, however its data is not designed to be reported as a composite quality score (see additional file 2). The CAT was designed to assess the impact of each individual item on the quality of the methodological procedures implemented in each paper. Prior to critical appraisal of the included articles, three papers were randomly selected and assessed independently by three reviewers using the purpose-built CAT. Disagreements were discussed to ensure that interpretation of the CAT items were consistent.

## Results

### Results from the search strategies

**Figure 2:**
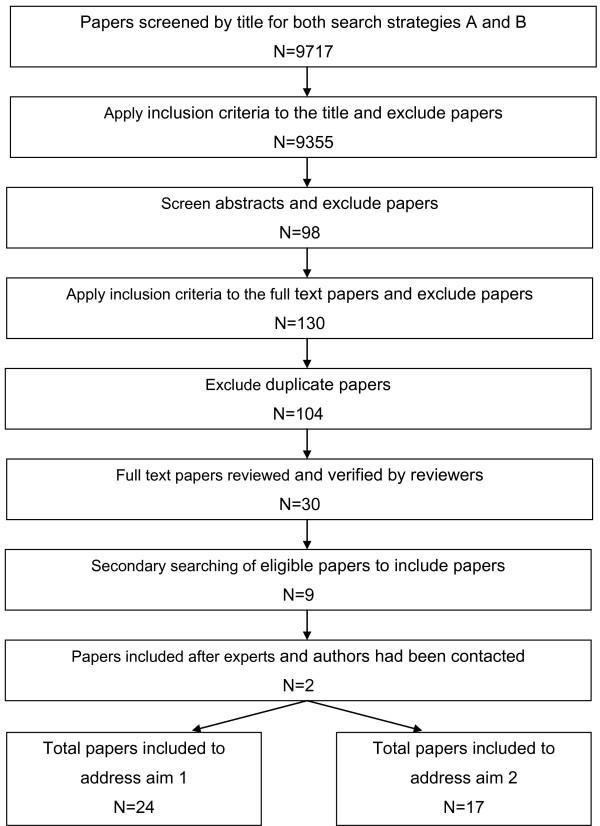
**Consort diagram to demonstrate the selection of papers**.

One hundred and thirty possible papers were considered, of which 30 papers were deemed to be eligible. Nine additional papers were identified after searching the reference lists of these papers. Two further papers were included after experts and authors had been contacted. Figure 2 provides a consort diagram to demonstrate the selection of papers.

### Volume of literature

Eighteen instruments were identified from the two literature searches, 15 from Search A, one from Search B and two from author contacts. The instruments are listed in the first column of Table 1, the papers addressing aim one appear in the second column and those addressing aim two are in the third column. Papers reporting these instruments, are identified by bold script if from strategy A, italics if from strategy B, normal script if from author search and with a * if from secondary searching. The Automatic Scoliosis Analyser System (Auscan) (Italy), the Elite system (Italy), the Optotrak 3020 (Canada), the Peak Motus (USA), the PosturePrint (Canada), the Qualysis Proreflex Motion Capture Unit system (Sweden), the Vicon 370 (England) and an Optoelectronic camera system (Canada) are optoelectronic analysis systems. The Fonar upright positional MRI (USA) uses magnetic resonance imaging. The INSPECK (Canada) is an optical 3D digitizer. The Lumbar Motion Monitor (LMM) (USA) is a electrogoniometer. The Metrecom (USA), the Articulated Arm for Computerized Surface Measurement (BACES) (Italy) and the Microscribe 3DX Digitizer (USA) are computerized electromechanical 3D digitizers. Rasterstereography is a photogrammetric method based on triangulation. The 3 Space Isotrak or Fastrak (USA) and the Electromagnetic tracking system (USA) are electromagnetic devices. The Zebris (Germany) is an ultrasound analysis system.

**Table 1:** Recent three-dimensional instruments used to measure static spinal posture

Instrument	Addresses Aim 1: Used to measure posture	Addresses Aim 2: Reports on psychometric properties	N
BACES	**D'Osualdo et al. 2002 **[41]		

AUSCAN	*Negrini et al. 2007 *[42]		

Electromagnetic tracking system	**Claus et al. 2009 **[43]		

Elite optoelectronic system	**Lissoni et al. 2001 **[44]**; Naslund et al. 2005 **[45]		

Inspek		Pazos et al. 2005* [35]; **Pazos et al. 2007 **[27]	2

Lumber Motion Monitor (LMM)	**Jang et al. 2007 **[46]		

FONAR Upright positional MRI	**Morl et al. 2006 **[47]; **Cargill et al. 2007 **[48]; **Lafon et al. 2010 **[49]		

Metrecom	Franklin et al. 1995* [50]; **Black et al. 1996 **[51]; **Gram et al. 1999 **[52]	Smidt et al. 1992* [22]; Norton et al. 1993* [38]	2

Microscribe 3DX Digitizer		Warren et al. 2005 [28]	1

Optoelectronic camera system	**Duong et al. 2009 **[53]		

Optotrak 3020	Rempel et al. 2007 [54]		

Peak Motus	**Straker et al. 2009 **[55]		

Postureprint		**Normand et al. 2002 **[37]; *Harrison et al. 2007 *[33]; **Janik et al. 2007 **[34]; *Normand et al. 2007 *[26]	4

Qualysis Proreflex Motion Capture Unit system	**Grip et al. 2007 **[56]**; Neiva et al. 2009 **[57]		

Rasterstereography		Stokes et al. 1988* [32]; **Hackenberg et al 2003a **[30]; *Hackenberg 2003b *[31]; Drerup et al. 1994* [23] and 1996* [24]	5

3 Space Isotrack/Fastrak	O' Sullivan et al. 2006* [58]; **Caneiro et al. 2010 **[59]; **Astfalck et al. 2010 **[60]	Pearcy et al. 1989* [36]	1

Vicon three-dimensional kinematic system	**Levine et al. 1996 [61}; Szeto et al. 2005 **[9]**; Skalli et al. 2006 **[62]	**Whittle et al. 1997 **[29]	1

Zebris CMS70P; Zebris CMS20	**Theisen et al. 2010 **[63]	**Geldhof et al. 2007 **[25]	1

N: number of papers addressing aim 2; Bold script: Papers from search A; Italic script: Papers from search B;*: Papers from secondary search; Normal script: Papers from author search

Seventeen papers reported on reliability and/or validity of the included instruments and were thus assessed to address Aim two (see Table 1 third column). One paper by Smidt et al. [22] reported on both reliability and validity, and was therefore reviewed as if it was two separate papers, due to the nature of this review. Drerup et al. [23] tested a new algorithm for processing data presented in a previous paper [24]. These papers were reviewed as if they were one paper, because the previous paper reported on the study procedure in more detail whereas the latter paper discussed the latest improvement made on the data processing procedure.

### Aim of the reliability studies

The aim of six studies was to test the reliability of a 3D instrument in assessing the spinal posture of humans [22,25-29].

### Aim of the validity studies

The aim of eleven studies was to test the validity of a 3D posture instrument. Four studies [23,30-32] used human subjects to measure 3D spinal posture and to compare the results with those obtained from a reference standard. The other seven studies either used mannequins [33-35], wooden wedges [36], a steel frame [22], parallelograms [37] or other objects with known parameters [38] to test the validity of an instrument that could be used to assess 3D spinal posture of humans in future.

### Study design for reliability and validity studies

The type of reliability and validity tested, as well as the time interval for the reliability studies and the reference standard for the validity studies, are reported in Table 2.

**Table 2:** The type and time interval for reliability studies and the type and reference standard for validity studies

Author	Type of reliability	Time interval	Type of validity	Reference standard
Stokes et al (1988)	N/A	N/A	Criterion-related validity	Stereoradiography

Pearcy et al (1989)	N/A	N/A	Concurrent validity	Precision optical inclinometer

Smidt et al (1992)	N/A	N/A	Concurrent validity	Not specified
	
	Intra- and interrater reliabIlity	On the same day	N/A	N/A

Norton et al (1993)	N/A	N/A	Concurrent validity	Type measure or ruler

Drerup et al (1996)	N/A	N/A	Criterion-related validity	Stereoradiography

Normand et al (2002)	N/A	N/A	Concurrent validity	Not specified

Hackenberg et al (2003a)	N/A	N/A	Criterion-related validity	Stereoradiography
				
Hackenberg et al (2003b)				

Pazos et al (2005)	N/A	N/A	Concurrent validity	Coordinate measuring machine

Harrison et al (2007) and Janik et al (2007)	N/A	N/A	Concurrent validity	Not specified

Whittle et al (1997)	Intrarater reliability	On the same day	N/A	N/A

Warren et al 2005	Intrarater reliability	One minute	N/A	N/A

Geldhof et al (2007)	Intrarater reliability	One week	N/A	N/A

Pazos et al (2007)	Test retest reliability	30 seconds	N/A	N/A

Normand et al (2007)	Intra- and interrater reliability	One day	N/A	N/A

N/A: Not Applicable

### Statistical analysis

Table 3 summarizes the statistical procedures implemented in the reliability and validity studies. Comparing the findings in this table with the types of reliability and validity testing reported in Table 2, highlights the variability in choice and application of statistical tests to assess the same constructs.

**Table 3:** Statistical procedures of the reliability and validity studies

Author	Statistical analysis
Stokes et al (1988)	• linear regression analysis and Pearson correlation coefficient ^®^

Pearcy et al (1989)	• means; estimate of error, regression analysis and ICC

Smidt et al (1992)	• Dunnett's comparison test

Norton et al (1993)	• Pearson product moment correlation coefficient ^® ^and repeated measures t test

Drerup et al (1996) and Hackenberg et al (2003a and b)	• Root mean square (RMS) deviations of the surface curves from the radiographic curves

Whittle et al (1997)	• ICC and Pearson correlation coefficient

Normand et al (2002)	• means, SD, SEM, 95% Confidence Intervals (CI) and mean differences

Pazos et al (2005)	• multiway ANOVA

Warren et al 2005	• Pearson correlation coefficient and ICC

Harrison et al (2007) and Janik et al (2007)	• error analyses of mean differences and SD

Geldhof et al (2007)	• ICC for test-retest reliability

Pazos et al (2007)	• bivariate ANOVA; typical error of measurement (TEM); 95% CI of the TEM; smallest detectable difference (SDD) and multivariate ANOVA

Normand et al (2007)	• mean absolute values of differences within examiner and between examiner measurements; ANOVA; Shapiro-Wilk test and SEM for conservative and liberal ICC methods

### Methodological Quality Appraisal

Table 4 reports the findings from the critical appraisal of the papers, related to reliability and validity testing.

**Table 4:** Summary of the methodological quality appraisal results of the studies (*n *= 17)

Authors	Item 1	Item 2	Item 3	Item 4	Item 5	Item 6	Item 7	Item 8	Item 9	Item 10	Item 11	Item 12	Item 13
Stokes et al (1988)	**√**	**x**	√	**n/a**	**n/a**	**n/a**	√	**n/a**	√	√	√	√	√

Pearcy et al (1989)	**n/a**	**x**	√	**n/a**	**n/a**	**n/a**	**n/a**	**n/a**	√	√	√	**n/a**	√

Smidt et al (1992) (validity)	**n/a**	**x**	**x**	**n/a**	**n/a**	**n/a**	**n/a**	**n/a**	**x**	√	**x**	**n/a**	√

Smidt et al (1992) (reliability)	**√**	√	**n/a**	√	√	**x**	**n/a**	√	**n/a**	√	**n/a**	**x**	√

Norton et al (1993)	**n/a**	**x**	**x**	**n/a**	**n/a**	**n/a**	**n/a**	**n/a**	√	√	√	**n/a**	**x**

Drerup et al (1994; 1996)	**x**	**x**	√	**n/a**	**n/a**	**n/a**	√	**n/a**	√	√	√	√	√

Whittle et al (1997)	**√**	**x**	**n/a**	**n/a**	**x**	**x**	**n/a**	√	**n/a**	√	**n/a**	√	√

Normand et al (2002)	**n/a**	**x**	**x**	**n/a**	**n/a**	**n/a**	**n/a**	**n/a**	**x**	√	**x**	**n/a**	√

Hackenberg et al (2003a)	**√**	**x**	√	**n/a**	**n/a**	**n/a**	√	**n/a**	√	**x**	√	**x**	√

Hackenberg et al (2003b)	**√**	**x**	√	**n/a**	**n/a**	**n/a**	√	**n/a**	√	**x**	√	**x**	√

Warren et al (2005)	**√**	**x**	**n/a**	**n/a**	**X**	**x**	**n/a**	√	**n/a**	√	**n/a**	**x**	√

Pazos et al. (2005)	**n/a**	**x**	√	**n/a**	**n/a**	**n/a**	**n/a**	**n/a**	√	√	√	**n/a**	√

Harrison et al (2007)	**n/a**	**x**	**x**	**n/a**	**n/a**	**n/a**	**n/a**	**n/a**	**x**	**√**	**x**	**n/a**	**√**

Janik et al (2007)	**n/a**	**x**	**x**	**n/a**	**n/a**	**n/a**	**n/a**	**n/a**	**x**	**√**	**x**	**n/a**	**√**

Geldhof et al (2007)	**√**	**x**	**n/a**	**n/a**	**√**	**x**	**n/a**	**√**	**n/a**	**√**	**n/a**	**√**	**√**

Pazos et al (2007)	**√**	**x**	**n/a**	**n/a**	**n/a**	**n/a**	**n/a**	**√**	**n/a**	**√**	**n/a**	**x**	**√**

Normand et al (2007)	**√**	**√**	**n/a**	**√**	**√**	**√**	**n/a**	**√**	**n/a**	**√**	**n/a**	**√**	**√**

Item 1: If human subjects were used, did the authors give a detailed description of the sample of subjects used to perform the (index) test?

Nine papers [22,25-32] scored "yes" because a detailed description of the sample characteristics was stated. Drerup et al. [23] scored "no" as the authors did not mention how their subjects were recruited and merely stated that only scoliosis patients were included. Seven papers [22,33-38] scored "not applicable" because these studies used inanimate objects.

Item 2: Did the authors clarify the qualification, or competence of the rater(s) who performed the (index) test?

Eleven validity studies [22,23,30-38] and four reliability studies [25,27-29] scored "no". The qualifications of the operators of the instruments were not reported, as there was no description of their past experience with operating these instruments. The reliability studies of Smidt et al. [22] and Normand et al. [26] scored "yes" as they stated that the operators were "familiar and competent" in its use.

Item 3: Was the reference standard explained?

Drerup et al. [23], Hackenberg et al. [30,31] and Stokes et al. [32] scored "yes" as they provided references for the methods used to digitize the radiographs. Pazos et al. [35] and Pearcy et al. [36] scored "yes" because the authors named and stated the accuracy of the instruments used as the reference standard. Norton et al. [38] scored "no" because the ruler or tape measure was inappropriately used as a reference standard for calculating 3D coordinates of a point in space. Harrison et al. [33], Janik et al. [34], Normand et al. [37] and Smidt et al. [22] scored "no" because the authors used an object with known 3D parameters as reference standards, but the methods to measure these 3D locations, angles or distances were not explained.

Item 4: If interrater reliability were tested, were raters blinded to the findings of other raters?

Normand et al. [26] and Smidt et al. [22] scored "yes" because subjects were evaluated separately by the different raters. Geldhof et al. [25], Warren et al. [28] and Whittle and Levine [29] only tested intrarater reliability and scored "not applicable". Pazos et al. [26] scored "not applicable" because no rater reliability was evaluated but instead test-retest reliability of the instrument, when using different postures, was evaluated.

Item 5: If intrarater reliability were tested, were raters blinded to their own prior findings of the test under evaluation?

Geldhof et al. [25], Normand et al. [26] and Smidt et al. [22] scored "yes" because the raters were sufficiently blinded to their own prior measurements as either repeated digitizing of the anatomical landmarks took place one week apart, all photographs were numbered and were not identifiable by subject name, occasion or characteristics, and no skin markings were made on subjects. Warren et al. [28] and Whittle and Levine [29] scored "no" because passive and skin markings respectively were placed only once on the subject and were not removed between repeated measurements. Pazos et al. [27] scored "not applicable" because they did not test rater reliability.

Item 6: Was the order of examination varied?

Normand et al. [26] scored "yes" because subjects were evaluated in random order. Warren et al. [28] and Whittle and Levine [29] scored "no" because repeated measurements were performed consecutively without changing the order of subjects during testing. Geldhof et al. [25] scored "no" as the order of testing was kept the same for the repeated measurements one week apart. Smidt et al. [22] scored "no" as insufficient information was provided. Pazos et al. [27] scored "not applicable" because no rater reliability was tested.

Item 7: If human subjects were used, was the time period between the reference standard and the index test short enough to be reasonably sure that the target condition did not change between the two tests?

Drerup et al. [23], Hackenberg et al. [30,31] and Stokes et al. [32] scored "yes" because the radiographs and the rasterstereographs were taken on the same day. The other seven articles [22,33-38] scored "not applicable" because inanimate objects which cannot deform with passage of time were used.

Item 8: Was the stability (or theoretical stability) of the variable being measured taken into account when determining the suitability of the time-interval between repeated measures?

Six papers scored "yes" because repeated measurements of posture were either taken on the same day [22,27-29] one week [25] or one day apart [26].

Item 9: Was the reference standard independent of the index test?

Seven papers [23,30-32,35,36,38] scored "yes" because the index test and the reference standard were independant instruments. Harrison et al. [33], Janik et al. [34], Normand et al. [37] and Smidt et al. [22] scored "no" due to insufficient information provided.

Item 10: Was the execution of the (index) test described in sufficient detail to permit replication of the test?

Nine validity [22,23,32-38] and six reliability papers [22,25-29] scored "yes" because clear descriptions of how the instruments were applied to the subjects or to the inanimate objects were provided. Hackenberg et al. [30,31] scored "no" as the authors did not explain how raterstereographs were performed on the subjects, nor did they provide any citations for the methodology.

Item 11: Was the execution of the reference standard described in sufficient detail to permit its replication?

Seven papers scored "yes" because clear descriptions of how the reference standard were used on the subjects [23,32] or on the inanimate objects [35,36,38] or citations for the methodology [30,31] were provided. Harrison et al. [33], Janik et al. [34], Smidt et al. [22] and Normand et al. [37] scored "no" for the reasoning provided for item 3.

Item 12: Were withdrawals from the study explained?

Drerup et al. [23], Geldhof et al. [25], Normand et al. [26], Stokes et al. [32] and Whittle and Levine [29], scored "yes" because the number of subjects who participated in the studies was reflected in the results sections of the studies. Hackenberg et al. [30,31] scored "no" as the authors did not explain why 48 instead of 52 and 24 instead of 25 subjects participated in the pre operative evaluations respectively. Pazos et al. [27], Warren et al. [28] and Smidt et al. [22] scored "no" due to insufficient information provided. Seven papers [22,33-38] scored "not applicable" because these studies used inanimate objects.

Item 13: Were the statistical methods appropriate for the purpose of the study?

All but one paper by Norton et al. [38] implemented appropriate statistical analysis and thus scored "no". Although the other sixteen papers reported appropriate statistical analysis only six papers [23,30,31,26,28] provided a justification or motivation for using their chosen statistical measures.

## Discussion

This review attempted to evaluate the quality of reporting of psychometric properties of 18 3D human posture measuring instruments. It identified a lack of well-documented studies testing the psychometric properties of these instruments, as papers describing the development of only eight instruments were found (see Table 1 column C). The review suggests that the PosturePrint and rasterstereography had relatively more psychometric testing than the other tools included in this review. However, the methodological quality of the testing procedures for all instruments was flawed, when considering the methodological criteria applied in this review.

### Rater qualification

Both reliability and validity studies should provide descriptions of the qualifications of the rater(s) used in the studies because the rater(s) professional background, expertise and prior training operating these instruments will affect psychometric property assessment. Appropriate training of raters is important to minimise measurement error, and to facilitate interpretation of findings. These factors should therefore be considered when interpreting study findings, and extrapolating them for applicability and generalisability to other clinical and research settings [39].

### Reference standard

Four studies, which used inanimate objects, did not identify the instruments used to obtain the known values of objects which provided the reference standard data. In order to test validity, it is important that the psychometric properties of the reference standard be known to confirm that the reference standard is suitable [39]. The most suitable non-invasive 3D reference standard for postural measurements has not been unanimously determined in this field of research. The validity studies that used humans also used stereoradiography as reference standard, as radiography remains the most accurate assessment for posture. This situation continues, even though there is a possible health risk for repeated X-ray exposure to healthy spines and organs [40].

Norton et al. [38] used a ruler or tape measure as a reference standard. The x, y, z coordinates obtained from the index test had to be mathematically transformed to distances between pairs of points before the reference data, obtained from the ruler or tape measure, could be used. It would have been better had these authors used a reference standard with known accuracy to measure 3D coordinates directly. The ruler or tape measure was also a poor reference standard to use when measuring the distance between pairs of points on the human skeleton.

### Blinding for intra- or interrater reliability

The repeated measurements by Geldhof et al. [25] were performed one week apart however the order of the subjects was fixed. Therefore this enhances the possibility for the raters to recall the test outcomes of the previous measurements and potentially incurs increased bias. Warren et al. [28] and Whittle and Levine [29] tested intrarater reliability however the marking of the anatomical landmarks was only undertaken once before repeated measurements were taken, without allowing for removal and replacement of the markers between repeated measurements. Both raters in these studies were not blinded to their previous measurements of the same subjects. Consequently this potentially introduced bias and compromised the quality of the studies and findings.

### Statistical analysis

Given the complexity of posture measurement and interpretation, no statistical strategy for psychometric property testing is without its disadvantages. Therefore it seems sensible to report the findings of two or more different statistical analysis approaches in order to validate findings [21]. This did not occur in any of the included papers. For example Pearcy et al. [36] used linear regression analysis to demonstrate that as the magnitude of the one variable increases so does the amount of error however there is no indication of a cut off value (e.g. 95% CI and SD) up to where the 3 Space Isotrak can be expected to accurately measure an angle.

As a variety of statistical measures were reported in this review, another method to improve reporting quality would be for authors to justify why they chose a particular statisical test, relevant to the purpose of testing. This would provide the reader with better insight into the results, and would perhaps guide future authors in choice, and interpretation of more appropriate statisical analysis. For example Norton et al. [38] used multiple analysis to determine whether there is agreement between measures. However Pearson product moment correlation only reports on the correlation between two different measurements and cannot quantify the amount of aggreement or indicate whether there is systematic error. Repeated t-tests are also inappropriate to test systematic differences, as this testing will inflate the type I error and compromise interpretation of significance.

### Limitations

One limitation to this review comes from our inability to retrieve potentially eligible papers from authors who failed to respond to email inquiries. It could be that there are other relevant instruments which have been adequately evaluated for reliability and validity, however these papers were not available despite using multiple search methods (database, internet and author searches).

## Conclusions

This review described 18 non-invasive ways of measuring static human 3D sitting or standing spinal posture, and the methodological procedures of testing reliability and validity of a subset of these instruments. The review concludes that further research into the reliability and validity testing of these instruments is required to improve the quality of reliability and validity evidence of posture-measuring instruments. Psychometric property testing should be improved by addressing rater qualification, clearer definitions of the reference standards, applying appropriate methodological procedures to enhance rater blinding and improving the quality of reported statistical analysis. By improving the methodological rigor of reliability and validity testing, it would consequently enhance users' confidence in the psychometric evidence of static human 3D sitting or standing spinal posture in clinical and research settings.

## Competing interests

The authors declare that they have no competing interests.

## Authors' contributions

YB and QL contributed to the conception and design of the study, YB acquired and analyzed the data and all authors YB, QL and KGS contributed to the interpretation of data, the drafting and critically appraising of the content of the manuscript. All authors read and approved the final manuscript.

## Pre-publication history

The pre-publication history for this paper can be accessed here:

http://www.biomedcentral.com/1471-2474/12/93/prepub

## References

[B1] Bullock-Saxton J Postural alignment in standing: A repeatability study Aust Physiother 1993 39 25 29 10.1016/S0004-9514(14)60466-9 25026059

[B2] Sheeran L Sparkes V Busse M Van Deursen R Preliminary study: reliability of the spinal wheel. A novel device to measure spinal postures applied to sitting and standing Eur Spine J 2010 19 995 1003 10.1007/s00586-009-1241-0 20013001 PMC2899977

[B3] Ariens GAM Bongers PM Douwes M Miedema MC Hoogendoorn WB Van der Wal G Bouter LM Van Mechelen W Are neck flexion, neck rotation, and sitting at work risk factors for neck pain? Results of a prospective cohort study Occup Environ Med 2001 58 200 207 10.1136/oem.58.3.200 11171934 PMC1740110

[B4] Auvinen BM Tammelin T Taimela S Zitting P Karppinen J Neck and shoulder pains in relation to physical activity and sedentary activities in adolescence Spine 2007 32 1038 1044 10.1097/01.brs.0000261349.94823.c1 17450080

[B5] Niemi SM Levoska S Kemila J Rekola K Keinanen-Kiukaanniemi SM Neck and shoulder symptoms and leisure time activities in high school students J of Orthop Sports and Phys Ther 1996 24 25 29 8807538 10.2519/jospt.1996.24.1.25

[B6] Prins Y Crous LC Louw QA A systematic review of posture and psychosocial factors as contributors to upper quadrant musculoskeletal pain in children and adolescents Physio Theory and Prac 2008 24 221 242 10.1080/09593980701704089 18574749

[B7] Ramos EMA James CA Bear-Lehman J Children's computer usage: Are they at risk of developing repetitive strain injury? Work 2005 25 143 154 16131744

[B8] Saarni L Nygard C Rimpela A Nummi T Kaukiainen A The working postures among schoolchildren - A controlled intervention study on the effects of newly designed workstations J of School Health 2007 77 240 247 10.1111/j.1746-1561.2007.00199.x 17430436

[B9] Szeto GPY Straker L O'Sullivan PB A comparison of symptomatic and asymptomatic office workers performing monotonous keyboard work-2: Neck and shoulder kinematics Man Ther 2005 10 281 291 10.1016/j.math.2005.01.005 15996890

[B10] Barrero M Hedge A Computer environment for children: A review of design issues Work 2002 18 227 237 12441563

[B11] Li G Buckle P Current techniques for assessing physical exposure to work-related musculoskeletal risks, with emphasis on posture-based methods Ergon 1999 42 674 695 10.1080/001401399185388 10327891

[B12] Hay O Hershkovitz I Rivlin E Spine curve modelling for quantitative analysis of spinal curvature31st Ann Int Conf of the IEEE EMBS 2009 Minneapolis, Minnesota, USA 6356 6359 10.1109/IEMBS.2009.5333263 19964161

[B13] Vieira ER Kumar S Working postures: A literature review J Occup Rehabil 2004 14 143 159 15074366 10.1023/b:joor.0000018330.46029.05

[B14] Vrtovec T Pernus F Likar B A review of methods for quantitative evaluation of spinal posture Eur Spine J 2009 18 593 607 10.1007/s00586-009-0913-0 19247697 PMC3233998

[B15] White SA Van den Broek NR Methods for assessing reliability and validity for a measurement tool: a case study and critique using WHO haemoglobin colour scale Stat Med 2004 23 1603 1619 10.1002/sim.1804 15122740

[B16] Karanicolas PJ Bhandari M Kreder H Moroni A Richardson M Walter SD Norman GR Guyatt GH Evaluating agreement: conducting a reliability study J Bone Joint Surg 2009 91 99 106 19411507 10.2106/JBJS.H.01624

[B17] Portney LG Watkins MP Foundations of clinical research: applications to practice 2009 3 New Jersy: Pearson Education

[B18] Brink H Fundamentals of research methodology for health care professionals 2006 2 Cape Town: Juta

[B19] Bannigan K Watson R Reliability and validity in a nutshell J Clin Nursing 2009 18 3237 3243 10.1111/j.1365-2702.2009.02939.x 19930083

[B20] Whiting P Rutjes AWS Reitsma JB Bossuyt PMM Kleijnen J The development of QUADAS: a tool for quality assessment of studies of diagnostic accuracy included in systematic reviews BMC Med Res Methodol 2003 3 25 37 10.1186/1471-2288-3-25 14606960 PMC305345

[B21] Lucas NP Macaskill P Irwig L Bogduk N The development of a quality tool for studies of diagnostic reliability (QAREL) J Clin Epidemiol 2010 63 854 861 10.1016/j.jclinepi.2009.10.002 20056381

[B22] Smidt GL McQuade KJ Wei S Evaluation of the Metrecom and its use in quantifying skeletal landmark locations J Orthop Sports and Phys Ther 1992 16 182 188 18796759 10.2519/jospt.1992.16.4.182

[B23] Drerup B Hierholzer E Assessment of scoliotic deformity from back shape asymmetry using an improved mathematical model Clin Biomech 1996 11 376 383 10.1016/0268-0033(96)00025-3 11415649

[B24] Drerup B Hierholzer E Back shape measurement using video rasterstereography and three-dimensional reconstruction of spinal shape Clin Biomech 1994 9 28 36 10.1016/0268-0033(94)90055-8 23916075

[B25] Geldhof E Cardon G De Bourdeaudhuij I Daneels L Coorevits P Vanderstraeten G De Clerq D Effects of back posture education on elementary schoolchildren's back function Eur Spine J 2007 16 829 839 10.1007/s00586-006-0199-4 16944227 PMC2200723

[B26] Normand MC Descarreaux M Harrison DD Harrison DE Perron DL Ferrantelli JR Janik TJ Three dimensional evaluation of posture in standing with the PosturePrint: an intra- and inter-examiner reliability study Chiropractic & Osteopathy 2007 15 15 25 10.1186/1746-1340-15-15 17892559 PMC2077332

[B27] Pazos V Cheriet F Dansereau J Ronsky J Zernicke RF Labelle H Reliability of trunk shape measurements based on 3-D surface reconstructions Eur Spine J 2007 16 1882 1891 10.1007/s00586-007-0457-0 17701228 PMC2223340

[B28] Warren JG Bettany-Saltikov J Van Schaik P Papastefanou S Evidence-based postural assessment for use in therapy and rehabilitation Int J of Ther and Rehabil 2005 12 527 532

[B29] Whittle MW Levine D Measurement of lumbar lordosis as a component of clinical gait analysis Gait and Posture 1997 5 101 107 10.1016/S0966-6362(96)01079-X

[B30] Hackenberg L Hierholzer E Potzl W Gotze C Liljenqvist U Rasterstereographic back shape analysis in idiopathic scoliosis after anterior correction and fusion Clin Biomech2003a 18 1 8 10.1016/S0268-0033(02)00165-1 12527240

[B31] Hackenberg L Hierholzer E Potzl W Gotze C Liljenqvist U Rasterstereographic back shape analysis in idiopathic scoliosis after posterior correction and fusion Clin Biomech2003b 18 883 889 10.1016/S0268-0033(03)00169-4 14580831

[B32] Stokes IAF Armstrong JG Moreland MS Spinal deformity and back surface asymmetry in idiopathic scoliosis J Orthop Res 1988 6 129 137 10.1002/jor.1100060117 3334733

[B33] Harrison DE Janik TJ Calliet R Harrison DD Normand MC Perron Ferrantelli JR Validation of a computer analysis to determine 3-D rotations and translations of the ric cage in upright posture from three 3-D digital images Eur Spine J 2007 16 213 218 10.1007/s00586-006-0081-4 16547756 PMC2200690

[B34] Janik TJ Harrison DE Calliet R Harrison DD Normand MC Perron DL Validity of a computer postural analysis to estimate 3-Dimensional rotations and translations of the head from three 2-Dimensional digital images J Manipul and Physiol Ther 2007 30 124 129 10.1016/j.jmpt.2006.12.005 17320733

[B35] Pazos V Cheriet F Song L Labelle H Dansereau J Accuracy assessment of human trunk surface 3D reconstructions from an optical digitising system Med & Biol Eng & Comp 2005 43 11 15 10.1007/BF02345117 15742714

[B36] Pearcy MJ Hindle RJ New method for the non-invasive three-dimensional measurement of human back movement Clin Biomech 1989 4 73 79 10.1016/0268-0033(89)90042-9 23915997

[B37] Normand MC Harrison DE Calliet R Black P Harrison DD Holland B Reliability and measurement error of the Biotonix Video Posture Evaluation system - Part I: Inanimate objects J of Manipul and Physiol Ther 2002 25 246 250 10.1067/mmt.2001.123169 12021743

[B38] Norton BJ Ellison JB Reliability and concurrent validity of the Metrecom for length measurements on inanimate objects Phys Ther 1993 73 266 274 8456145 10.1093/ptj/73.4.266

[B39] Bossuyt PM Reitsma JB Bruns DE Gatsonis CA Glasziou PP Irwig LM Moher D Rennie D De Vet HCW Lijmer JG The STARD Statement for reporting studies of diagnostic accuracy: Explanation and elaboration Clin Chem 2003 49 7 18 10.1373/49.1.7 12507954

[B40] Wagner M Bowing B Deimling M Rascher W Rupprecht T Low field thoracic MRI - a fast and radiation free routine imaging modality in children Magnetic Resonance Imaging 2001 19 975 983 10.1016/S0730-725X(01)00417-9 11595369

[B41] D'Osualdo F Schierano S Soldano M Isola M New tridimensional approach to the evaluation of the spine through surface measurement: the BACES system J Med Eng & Technol 2002 26 95 105 12350275 10.1080/03091900110114389

[B42] Negrini S Negrini A Postural effects of symmetrical and asymmetrical loads on the spines of schoolchildren Scoliosis 2007 2 8 14 10.1186/1748-7161-2-8 17620121 PMC1971247

[B43] Claus AP Hides JA Moseley GL Hodges P Is 'ideal' sitting posture real?: Measurement of spinal curves in four sitting postures Man Ther 2009 14 404 408 10.1016/j.math.2008.06.001 18793867

[B44] Lissoni A Caimmi M Rossini M Terenghi L Three-dimensional analysis of the sitting posture Europa Medicophysica 2001 37 101 109

[B45] Naslund A Jesinkey K Sundelin G von Wendt L Hirschfeld H Effects of dynamic ankle-foot orthoses on standing in children with severe spastic diplegia Int J Ther and Rehabil 2005 12 200 207

[B46] Jang R Karwowski W Quesadas PM Rodrick D Sherehiy B Cronin SN Layer JK Biomechanical evaluation of nursing tasks in a hospital setting Ergonomics 2007 50 1835 1855 10.1080/00140130701674661 17972205

[B47] Morl F Blickhan R Three-dimensional relation of skin markers to lumbar vertebrae of healthy subjects in different postures measured by open MRI Eur Spine 2006 15 742 751 10.1007/s00586-005-0960-0 PMC3489463 16047207

[B48] Cargill SC Pearcy M Darrell Barry M Three-dimensional lumbar spine postures measured by magnetic resonance imaging reconstruction Spine 2007 32 1242 1248 10.1097/01.brs.0000263404.66369.a5 17495783

[B49] Lafon Y Smith FW Beillas P Combination of a model-deformation method and a positional MRI to quantify the effects of posture on the anatomical structures of the trunk J of Biomech 2010 43 1269 1278 10.1016/j.jbiomech.2010.01.013 20226466

[B50] Franklin ME Chenier TC Brauninger L Cook H Harris S Effect of positive heel inclination on posture J of Orthop Sports and Phys Ther 1995 21 94 99 7711763 10.2519/jospt.1995.21.2.94

[B51] Black KM McClure P Polansky M The influence of different sitting positions on cervical and lumbar posture Spine 1996 21 65 70 10.1097/00007632-199601010-00015 9122765

[B52] Gram M Hasan Z The spinal curve in standing and sitting posture in children with idiopathic scoliosis Spine 1999 24 169 177 10.1097/00007632-199901150-00019 9926389

[B53] Duong L Mac-Thiong J Labelle H Real time non-invasive assessment of external trunk geometry during surgical correction of adolescent idiopathic scoliosis Scoliosis 2009 4 5 15 10.1186/1748-7161-4-5 19239713 PMC2651122

[B54] Rempel D Barr A Brafman D Young E The effect of six keyboard designs on wrist and forearm postures Appl Ergon 2007 38 293 298 10.1016/j.apergo.2006.05.001 16806042

[B55] Straker LM Maslen B Burgess-Limerick R Pollock C Children have less variable postures and muscle activities when using new electronic information technology compared with old paper-based information technology J of Electromyography and Kinesiology 2009 9 e132 e143 10.1016/j.jelekin.2007.11.011 18248823

[B56] Grip H Sundelin G Gerdle B Karlsson JS Variations in the axis of motion during head repositioning - A comparison of subjects with whiplash-associated disorders or non-specific neck pain and healthy controls Clin Biomech 2007 22 865 873 10.1016/j.clinbiomech.2007.05.008 17619066

[B57] Neiva PD Kirkwood RN Godinho R Orientation and position of head posture, scapula and thoracic spine in mouth-breathing children Int J of Pediatric Otorhinolaryngology 2009 73 227 236 10.1016/j.ijporl.2008.10.006 19056131

[B58] O'Sullivan P Dankaerts W Burnett A Straker L Bargon G Moloney N Perry M Tsang S Lumbopelvic kinematics and trunk muscle activity during sitting on stable and unstable surfaces J of Orthop Sports and Phys Ther 2006 36 19 25 16494070 10.2519/jospt.2006.36.1.19

[B59] Caneiro JP O'Sullican P Burnett A Barach A O'Neil D Tveit O Olafsdottir K The influence of different sitting postures on head/neck posture and muscle activity Man Ther 2010 15 54 60 10.1016/j.math.2009.06.002 19643658

[B60] Astfalck RG O'Sullivan P Straker LM Smith AJ Burnett A Caneiro JP Sitting postures and trunk muscle activity in adolescent with and without nonspecific chronic low back pain Spine 2010 35 1387 1395 20195206 10.1097/BRS.0b013e3181bd3ea6

[B61] Levine D Whittle MW The effects of pelvic movement on lumbar lordosis in the standing position J of Orthop Sports and Phys Ther 1996 24 130 135 8866271 10.2519/jospt.1996.24.3.130

[B62] Skalli W Zeller RD Miladi L Bourcereau G Savidan M Lavaste F Dubousset J Importance of pelvic compensation in posture and motion after posterior spinal fusion using CD instrumentation for idiopathic scoliosis Spine 2006 31 E359 366 10.1097/01.brs.0000219402.01636.87 16721280

[B63] Theisen C van Wagensveld A Timmesfeld N Efe T Heyse TJ Fuchs-Winkelmann S Schofer MD Co-occurence of outlet impimgement syndrome of the shoulder and restricted range of motion in the thoracic spine - a prospective study with ultrasound-based motion analysis BMC Musculoskeletal Disorders 2010 11 135 144 10.1186/1471-2474-11-135 20587014 PMC2903509

